# Supplementary material for: Seeding food security: Overcoming barriers to quality potato seed adoption among smallholders in Kenya
Source: PLoS One. 2026 May 8;21(5):e0346796. doi: 10.1371/journal.pone.0346796 (PMC13155629; doi:10.1371/journal.pone.0346796)
Supplement: S1 Table — (DOCX) [file pone.0346796.s001.docx]

**S1 Table**. Comparison of Heckman with OLS estimates of the outcome equation (sensitivity analysis)

| **Variables** | **Coefficient** | **p** |
| --- | --- | --- |
| Gender of household head | 0.106 (0.081) | 0.192 |
| Age of household head | -0.007^**^(0.004) | 0.035 |
| Primary education | 0.142 (0.374) | 0.704 |
| Secondary education | 0.085 (0.364) | 0.815 |
| Tertiary education | -0.052(0.379) | 0.891 |
| Household size | -0.006 (0.027) | 0.840 |
| Total land size | 0.733^***^ (0.030) | 0.000 |
| Total household income | 0.077^*^(0.046) | 0.092 |
| Extension access | -0.071(0.093) | 0.449 |
| Credit access | 0.324^***^(0.113) | 0.005 |
| High-value market access | 0.419^*^(0.243) | 0.086 |
| Digital information | 0.163^*^(0.092) | 0.076 |
| Potato contract | -0.555^**^(0.234) | 0.019 |
| Registered farmer | 0.116 (0.113) | 0.309 |
| Distance road | 0.040^*^(0.020) | 0.053 |
| Distance seed source | 0.016 (0.015) | 0.292 |
| Distance market | -0.030^**^(0.012) | 0.012 |
| Manure access | 0.037 (0.088) | 0.672 |
| Livestock portfolio | 0.044 (0.037) | 0.233 |
| Constant | -0.840 (0.567) | 0.140 |
| F(19,219) | 53.39 |  |
| Adjusted R^2^ | 0.8070 |  |
| Prob>F | 0.000 |  |
| N | 239 |  |

**Notes:** Standard errors in parentheses

^*^ *p* < 0.1, ^**^ *p* < 0.05, ^***^ *p* < 0.01
