# Supplementary material for: Seeding food security: Overcoming barriers to quality potato seed adoption among smallholders in Kenya
Source: PLoS One. 2026 May 8;21(5):e0346796. doi: 10.1371/journal.pone.0346796 (PMC13155629; doi:10.1371/journal.pone.0346796)
Supplement: S2 Table — (DOCX) [file pone.0346796.s002.docx]

**S2 Table**. Over-identification checks of the instruments used in the selection equation

|  | **Selection model (probit) n=541** | **Outcome model (OLS) n=239** |
| --- | --- | --- |
| **Variables** | Coef. | Coef. |
| Gender of household head | 0.102(0.127) | 0.102(0.088) |
| Age of household head | -0.016^***^(0.005) | -0.011^**^(0.005) |
| Primary education | 0.711^*^(0.401) | 0.321(0.381) |
| Secondary education | 1.118^***^(0.404) | 0.391(0.403) |
| Tertiary education | 1.097^**^(0.436) | 0.234(0.411) |
| Household size | -0.015(0.038) | -0.017(0.028) |
| Total land size | 0.081(0.055) | 0.749^***^(0.033) |
| Total household income | 0.131^*^(0.070) | 0.125^**^(0.055) |
| Extension access | 0.327^**^(0.143) | 0.038(0.113) |
| Credit access | -0.327^*^(0.179) | 0.274^**^(0.130) |
| Access high value-market | -0.249(0.383) | 0.364(0.256) |
| Digital information | -0.054(0.138) | 0.159^*^(0.096) |
| Potato contract | 0.398(0.539) | -0.410(0.264) |
| Registered as a farmer | -0.023(0.191) | 0.158(0.127) |
| Distance road | -0.066^**^(0.029) | 0.024(0.023) |
| Distance seed source | 0.030(0.023) | 0.017(0.015) |
| Distance market | -0.023(0.017) | -0.035^***^(0.013) |
| Manure access | 0.199(0.148) | 0.123(0.110) |
| Livestock portfolio | 0.193^***^(0.056) | 0.114^**^(0.054) |
| Membership in farmers' organization | 0.397^**^(0.183) | -0.075(0.128) |
| Access to input subsidies | 0.441^***^(0.134) | 0.085(0.118) |
| Nyandarua | -0.218(0.168) |  |
| Nakuru | -0.601^***^(0.188) |  |
| Constant | -2.020^**^(0.797) | -1.976^**^(0.888) |
| lambda | 0.509^*^(0.306) |  |
| Rho | 0.749 |  |
| Sigma | 0.680 |  |
| N | 541 | 239 |

**Notes**: Standard errors in parentheses

^*^ *p* < 0.1, ^**^ *p* < 0.05, ^***^ *p* < 0.01
