# Supplementary material for: Seeding food security: Overcoming barriers to quality potato seed adoption among smallholders in Kenya
Source: PLoS One. 2026 May 8;21(5):e0346796. doi: 10.1371/journal.pone.0346796 (PMC13155629; doi:10.1371/journal.pone.0346796)
Supplement: S3 Table — (DOCX) [file pone.0346796.s003.docx]

S3 Table. VIF test for multicollinearity among independent variables in the outcome equation

| **Variables** | **VIF** |
| --- | --- |
| Gender of household head | 1.07 |
| Age of household head | 1.26 |
| Primary education | 16.20 |
| Secondary education | 21.72 |
| Tertiary education | 14.67 |
| Household size | 1.18 |
| Total land size | 1.52 |
| Total household income | 1.40 |
| Extension access | 1.42 |
| Credit access | 1.22 |
| High-value market access | 1.12 |
| Digital information | 1.26 |
| Potato contract | 1.19 |
| Registered farmer | 1.29 |
| Distance road | 1.58 |
| Distance seed source | 1.81 |
| Distance market | 1.74 |
| Manure access | 1.26 |
| Livestock portfolio | 1.21 |
| Mean VIF | 3.92 |
