# Supplementary material for: Seeding food security: Overcoming barriers to quality potato seed adoption among smallholders in Kenya
Source: PLoS One. 2026 May 8;21(5):e0346796. doi: 10.1371/journal.pone.0346796 (PMC13155629; doi:10.1371/journal.pone.0346796)
Supplement: S4 Table — (DOCX) [file pone.0346796.s004.docx]

S4 Table. Comparison of Heckman outcome coefficients with marginal effects from alternative models for robustness checks

|  | **Adoption (Probit)** | **Intensity**  **(Truncated model)** | **Intensity**  **(OLS)** | **Intensity (Heckman)** |
| --- | --- | --- | --- | --- |
|  | **(1)** | **(2)** | **(3)** | **(4)** |
| **Variables** | **ME** | **ME** | **ME** | **Coef.** |
| Gender of household head | 0.031(0.038) | 0.208 (0.152) | 0.106(0.081) | 0.109 (0.082) |
| Age of household head | -0.005***(0.002) | -0.013^*^(0.007) | -0.007^**^(0.004) | -0.010^***^(0.004) |
| Primary education | 0.187**(0.087) | 0.261(0.836) | 0.142(0.374) | 0.289 (0.368) |
| Secondary education | 0.316***(0.090) | 0.338(0.816) | 0.085(0.364) | 0.336 (0.373) |
| Tertiary education | 0.309***(0.104) | 0.046(0.833) | -0.052(0.379) | 0.191(0.387) |
| Household size | -0.004(0.011) | 0.031(0.051) | -0.006(0.027) | -0.014 (0.028) |
| Total land size | 0.024 (0.016) | 0.828^***^(0.045) | 0.733^***^(0.030) | 0.750^***^(0.032) |
| Total household income | 0.039*(0.021) | 0.260^***^(0.091) | 0.077^*^(0.046) | 0.119^**^(0.051) |
| Extension access | 0.098**(0.043) | 0.098(0.172) | -0.071(0.093) | 0.024 (0.105) |
| Credit access | -0.098*(0.053) | 0.556^***^(0.189) | 0.324^***^(0.113) | 0.270^**^(0.118) |
| Access high value-market | -0.075(0.115) | 0.406(0.384) | 0.419^*^(0.243) | 0.349 (0.249) |
| Digital information | -0.016(0.041) | 0.428^**^(0.187) | 0.163^*^(0.092) | 0.155^*^(0.092) |
| Potato contract | 0.119(0.162) | -0.853^**^(0.349) | -0.555^**^(0.234) | -0.455*(0.251) |
| Registered as a farmer | -0.007(0.057) | -0.003(0.208) | 0.116(0.113) | 0.118 (0.115) |
| Distance road | -0.020**(0.009) | 0.072^**^(0.036) | 0.040^*^(0.020) | 0.027 (0.115) |
| Distance seed source | 0.009(0.007) | 0.026(0.026) | 0.016(0.015) | 0.017(0.015) |
| Distance market | -0.007(0.005) | -0.035(0.023) | -0.030^**^(0.012) | -0.034^***^ (0.012) |
| Manure access | 0.060(0.044) | -0.060(0.169) | 0.037(0.088) | 0.119 (0.097) |
| Livestock portfolio | 0.058***(0.016) | 0.085(0.066) | 0.044(0.037) | 0.095^**^ (0.045) |
| Membership in farmers' organization | 0.119**(0.054) |  |  |  |
| Access to input subsidies | 0.132***(0.039) |  |  |  |
| Nyandarua | -0.070(0.054) |  |  |  |
| Nakuru | -0.188***(0.060) |  |  |  |
| N | 541 | 239 | 239 | 239 |

**Notes**: Standard errors in parentheses; ^*^ *p* < 0.1, ^**^ *p* < 0.05, ^***^ *p* < 0.01; ME is marginal effect
