# Supplementary material for: Seeding food security: Overcoming barriers to quality potato seed adoption among smallholders in Kenya
Source: PLoS One. 2026 May 8;21(5):e0346796. doi: 10.1371/journal.pone.0346796 (PMC13155629; doi:10.1371/journal.pone.0346796)
Supplement: S5 Table — (DOCX) [file pone.0346796.s005.docx]

S5 Table. Sensitivity analysis using Rosenbaum bounds for HDD and FCS

| **Gamma** | **Sig+** | **Sig-** | **t-hat+** | **t-hat-** |
| --- | --- | --- | --- | --- |
| HDDS |  |  |  |  |
| 1 | 0 | 0 | 8 | 8 |
| 1.5 | 0 | 0 | 7.5 | 8 |
| 2 | 0 | 0 | 7.5 | 8.5 |
| FCS |  |  |  |  |
| 1 | 0 | 0 | 75.5 | 75.5 |
| 1.5 | 0 | 0 | 72 | 78.5 |
| 2 | 0 | 0 | 69.75 | 80.75 |

**Notes**: t-hat+: upper bound Hodges-Lehmann point estimate, t-hat-: lower bound Hodges-Lehmann point estimate; sig+: upper bound significance level, sig-: lower bound significance level
