# Supplementary figures and images for: Seeding food security: Overcoming barriers to quality potato seed adoption among smallholders in Kenya
Source: PLoS One. 2026 May 8;21(5):e0346796. doi: 10.1371/journal.pone.0346796 (PMC13155629; doi:10.1371/journal.pone.0346796)

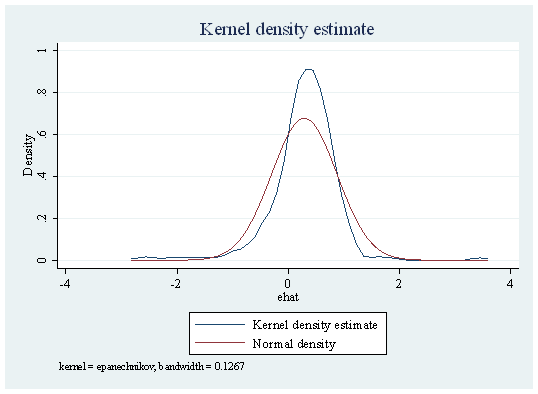

Supplement: S1 Fig — (TIF) [file pone.0346796.s006.tif]

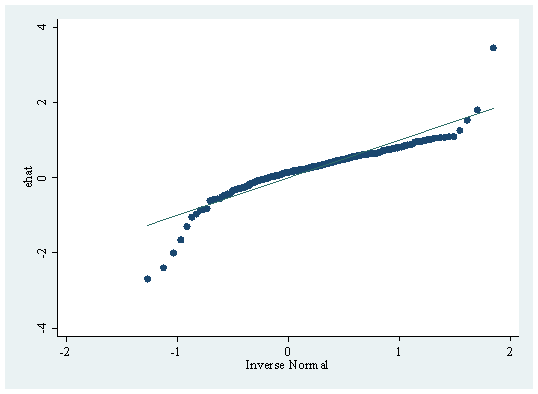

Supplement: S2 Fig — (TIF) [file pone.0346796.s007.tif]

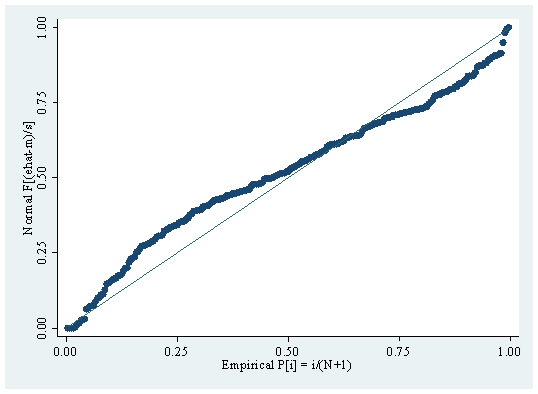

Supplement: S3 Fig — (TIF) [file pone.0346796.s008.tif]
