# Supplementary material for: Seeding food security: Overcoming barriers to quality potato seed adoption among smallholders in Kenya
Source: PLoS One. 2026 May 8;21(5):e0346796. doi: 10.1371/journal.pone.0346796 (PMC13155629; doi:10.1371/journal.pone.0346796)
Supplement: Attachment — Submitted filename: Response to Reviewers.docx [file pone.0346796.s010.docx]

**Response to the Editor and Reviewers**

Dear Sir/Madam,

We sincerely thank the Editor and the Reviewers for their careful evaluation of our manuscript and for the constructive comments and suggestions provided. We have revised the manuscript accordingly and believe that the changes have strengthened its clarity, rigor, and overall quality. Below, we provide a detailed, point-by-point response to each comment. All revisions have been incorporated into the manuscript and are highlighted in the marked-up version.

**Table 1: General editor’s comments**

|  | **Editor’s comments** | **Response** | **Page and line number** |
| --- | --- | --- | --- |
| 1 | Please ensure that your manuscript meets PLOS ONE's style requirements, including those for file naming. | We have revised the manuscript to fully comply with PLOS ONE formatting requirements, including font size, line numbering, heading styles, and file naming | Entire manuscript |
| 2 | In the ethics statement in the Methods, you have specified that verbal consent was obtained. Please provide additional details regarding how this consent was documented and witnessed, and state whether this was approved by the IR | We have revised the ethics statement to provide additional details on how verbal consent was obtained, documented, and approved by the Institutional Research Ethics Committee, in line with PLOS ONE requirements. | Page 10 line 211-221 |
| 3 | We note that the grant information you provided in the ‘Funding Information’ and ‘Financial Disclosure’ sections do not match. When you resubmit, please ensure that you provide the correct grant numbers for the awards you received for your study in the ‘Funding Information’ section. | We have ensured the grant information is reported correctly in both the ‘funding information section’ |  |
| 4 | When completing the data availability statement of the submission form, you indicated that you will make your data available on acceptance. Please note that, though access restrictions are acceptable now, your entire data will need to be made freely accessible if your manuscript is accepted for publication. | The dataset underlying this study has been deposited in Figshare. It is currently under embargo but is available to reviewers via the following private link: <https://figshare.com/s/4cc96e41a8ebc69d8162>. Upon publication, the dataset will be made publicly accessible under DOI: 10.6084/m9.figshare.31331608. | Page 44 line 830-832 |
| 5 | We note that Figure 2 in your submission contain map images which may be copyrighted. All PLOS content is published under the Creative Commons Attribution License (CC BY 4.0), which means that the manuscript, images, and Supporting Information files will be freely available online, and any third party is permitted to access, download, copy, distribute, and use these materials in any way, even commercially, with proper attribution. For these reasons, we cannot publish previously copyrighted maps or satellite images created using proprietary data, such as Google software (Google Maps, Street View, and Earth).  We require you to either (1) present written permission from the copyright holder to publish these figures specifically under the CC BY 4.0 license, or (2) remove the figures from your submission: | We have removed Figure 2 to avoid potential copyright concerns related to map imagery and instead provided a detailed textual description of the study area in the Methods section. | Page 9 line 197 |
| 6 | If the reviewer comments include a recommendation to cite specific previously published works, please review and evaluate these publications to determine whether they are relevant and should be cited. There is no requirement to cite these works unless the editor has indicated otherwise. | No new citation was recommended by reviewers |  |
| 7 | Please review your reference list to ensure that it is complete and correct. If you have cited papers that have been retracted, please include the rationale for doing so in the manuscript text, or remove these references and replace them with relevant current references. Any changes to the reference list should be mentioned in the rebuttal letter that accompanies your revised manuscript. If you need to cite a retracted article, indicate the article’s retracted status in the References list and also include a citation and full reference for the retraction notice. | We have thoroughly reviewed the entire reference list to ensure completeness, correctness, and consistency with PLOS ONE formatting guidelines. We confirm that none of the cited articles have been retracted. Minor formatting inconsistencies have been corrected in the revised manuscript. | Page 45-56 |

**Specific reviewers’ comments**

Table 2: Specific reviewers’ comments

|  | **Reviewer #1**  **Comments** | |  |
| --- | --- | --- | --- |
|  | **Reviewer comments** | **Response** | **Page and line number** |
| 1 | **Clarity on “quality seed” classification**  Quality seed includes certified seed, rapid multiplication, and “known-source” seed not formally inspected. This category is broad. The paper should clarify:  -how many farmers used certified vs. non-certified quality seeds, and  -whether impacts differ by seed type. | -We have revised the methods and results sections to clarify that “quality seed” is defined as seed obtained from recognized formal or semi-formal sources, including certified seed, RMT seed, and known-source seed from recognized seed multipliers. Farmers who reported using any of these seed types were classified as adopters of quality seed, while those who used their own saved seed, borrowed seed from friends, or obtained seed from local markets were classified as non-adopters of quality seed.  -We have also reported the numbers of adopters and non-adopters of quality seed.  -The impact analysis used a binary variable (quality seed adopters vs. non-adopters), and the results showed a significant difference in impact between adopters and non-adopters of quality seeds. | Page 12 line 247-254,  Page 20 line 436-438,  Page 34 line 620-626 |
| 2 | **Need stronger explanation of intensity variable:** Intensity is defined as land allocated to quality seed, but later model output mentions “acres planted” rather than “proportion” — this needs consistency and clearer measurement | We have revised the methods section to state that intensity is measured as the total land area (in acres) allocated to quality seeds among adopters, rather than as a proportion of total farm size. We have ensured consistent terminology throughout the manuscript and removed any references to “proportion” to avoid ambiguity. The results section has also been revised for clarity. | Page 12 line 259-261 |
| 3 | **Some factors have unexpected** **signs (credit variable)**  Credit access negatively influences adoption but increases intensity. This is explained, but it would be stronger if supported with more qualitative evidence or discussion of how credit is used (school fees, health, etc.). | We have strengthened the discussion section to provide a more theoretically grounded explanation, supported by relevant empirical literature showing that access to credit does not necessarily increase technology adoption but can facilitate scaling up among adopters. Specifically, we now argue that initial adoption decisions may depend more strongly on risk perception, agronomic uncertainty, and competing household priorities rather than liquidity alone. Moreover, credit may not automatically translate into adoption if farmers perceive using quality seed as risky. However, once adoption occurs, the scaling up (intensity) becomes more capital-dependent, explaining the positive association between credit and land allocated to quality seed. Additional literature has been cited to support this interpretation. | Page 39 line 727-737  Page 59 reference 82 and 84 |
| 4 | **Sustainability and long-term adoption not addressed**:  The study itself acknowledges it does not examine when adoption occurred or whether farmers will continue using quality seed. This is important for policy, since seed adoption may be seasonal or temporary. | We have expanded the policy implications, emphasizing the need for interventions that promote sustained seed use, continuous access to quality seed, and strengthened extension follow-up.  We have now clarified that the cross-sectional nature of our data limits our ability to determine whether adoption is continuous or temporary.  We have suggested that future research should employ panel or duration models to assess the sustainability of adoption. For example, they could examine the duration between awareness and adoption, and the persistence of quality seed use over time, to better understand sustainability dynamics | Page 42, line 801-806  Page 43 line 710-818 |
| **Reviewer #2 comments** | | | |
|  | **Comment** | **Response** | Page and line number |
| **1** | General comments: The manuscript addresses a critical issue regarding food security and agricultural productivity in Sub-Saharan Africa. The research question is clearly defined, and the study design is technically sound. | We thank the reviewer for the positive evaluation of the study design and analytical approach. |  |
| 2 | There is a critical compliance issue regarding the Data Availability Policy that must be resolved before publication. | Regarding the data Availability policy, the dataset has now been deposited in Figshare. The files are currently under embargo and accessible to reviewers via a private link provided to the journal (https://figshare.com/s/4cc96e41a8ebc69d8162). The dataset will be publicly released upon acceptance of the manuscript. The data availability statement in the manuscript has been updated accordingly. | Page 44 line 830-832 |
| 3 | Additionally, there are minor formatting and grammatical errors that require correction | All identified formatting and grammatical issues have been corrected in the entire manuscript |  |
| 4. | **Methodology and Analysis**  Model Justification: The justification for using the Heckman two-stage model over Tobit or Double Hurdle models is well-reasoned and supported by the literature. | We appreciate the reviewer’s positive assessment of our model justification. No changes were required for this section |  |
| 5 | **Instrument Validity:** The exclusion restrictions and pre-estimation checks suggest the statistical approach is rigorous. | We thank the reviewer for recognizing the rigor of the identification strategy and diagnostic tests. No changes were required in this section. |  |
| 6 | **Robustness Checks:**  The use of three matching algorithms and Rosenbaum bounds strengthens the validity of the findings. | We appreciate this observation. No changes were required in this section |  |
| 7 | **Results and Interpretation**: Credit Access Paradox: The finding that credit access negatively influences initial adoption but positively influences intensity of use is fascinating. The discussion suggests this may be due to the diversion of funds to non-agricultural needs. It would be valuable if the authors could expand on this in the Discussion section—perhaps suggesting that initial adoption relies more on risk perception than liquidity, whereas scaling up (intensity) is purely capital-dependent. | We have strengthened the discussion section to provide a more theoretically grounded explanation, supported by relevant empirical literature showing that access to credit does not necessarily increase technology adoption but can facilitate scaling up among adopters. Specifically, we now argue that initial adoption decisions may depend more strongly on risk perception, agronomic uncertainty, and competing household priorities rather than liquidity alone. Moreover, credit may not automatically translate into adoption if farmers perceive using quality seed as risky. However, once adoption occurs, the scaling up (intensity) becomes more capital-dependent, explaining the positive association between credit and land allocated to quality seed. | Page 39 line 727-737 |
| 8 | Formatting of Units: There are several instances where LaTeX-style formatting codes have remained in the text (e.g., Page 13, line 131: $8~tons/ha$). Please strip these formatting tags to ensure the text is clean standard English. | All residual LaTeX formatting tags have been removed from the manuscript. Units are now presented in clean, standard English format (e.g., “8 tons/ha”). | Page 6 line 131-132  Page 31 line 556 |
| 9 | **Minor Corrections:** Page 19, Lines 417-418: The phrase "… can be able to weigh the benefits..." is tautological. Please revise to "are able to weigh" or "can weigh" . | The sentence has been revised to read: “Young and more educated farmers are able to weigh the benefits of quality seed adoption more effectively.” | Page 20 line 439-440 |
| 10 | Page 35, Line 639: "These findings align with misconceptions... reported by [37] and partly explain why..." strictly speaking, the subject "misconceptions" is plural, but if the subject is the "findings" (plural), "explain" is correct. However, if referring to the "study" or "report", check for subject-verb agreement. | We have reviewed and corrected the sentence to ensure proper subject–verb agreement for clarity and grammatical accuracy. | Page 36 line 666-669 |
| 11 | Page 53 (References): There is a typo in the supporting information list: "Rosesnbaum bounds" should be corrected to "Rosenbaum bounds". | This has been corrected to “Rosenbaum bounds” in the supporting information section. | Page 56 (Reference) |
| 12 | Consistency: Ensure consistent capitalization of "Sub-County" throughout the manuscript. | We have carefully reviewed the manuscript and ensured consistent capitalization of “Sub-County” throughout the manuscript | Page 10 line 223  Page 36 line 662 |
